# The Role of Oxidative Stress and Cellular Senescence in the Pathogenesis of Metabolic Associated Fatty Liver Disease and Related Hepatocellular Carcinoma

**DOI:** 10.3390/antiox12061269

**Published:** 2023-06-14

**Authors:** Nikolaos-Andreas Anastasopoulos, Antonia V. Charchanti, Alexandra Barbouti, Eleftheria M. Mastoridou, Anna C. Goussia, Anastasia D. Karampa, Dimitrios Christodoulou, Georgios K. Glantzounis

**Affiliations:** 1 HPB Unit, Department of Surgery, Faculty of Medicine, School of Health Sciences, University of Ioannina, 45110 Ioannina, Greece; nata_kar2007@yahoo.gr; 2 Department of General Surgery, Croydon University Hospital, Croydon Health Services NHS Trust, London CR7 7YE, UK; 3 Department of Anatomy-Histology-Embryology, Faculty of Medicine, School of Health Sciences, University of Ioannina, 45110 Ioannina, Greece; acharcha@uoi.gr (A.V.C.); abarbout@uoi.gr (A.B.); md06474@uoi.gr (E.M.M.); 4 Department of Pathology, Faculty of Medicine, School of Health Sciences, University of Ioannina, 45110 Ioannina, Greece; agoussia@uoi.gr; 5 Department of Gastroenterology, Faculty of Medicine, School of Health Sciences, University of Ioannina, 45110 Ioannina, Greece; dchristo@uoi.gr

**Keywords:** MAFLD, hepatocellular carcinoma, oxidative stress, premature senescence, replicative senescence, mitochondrial dysfunction, lipotoxicity, senotherapeutics

## Abstract

Hepatocellular carcinoma (HCC) represents a worryingly increasing cause of malignancy-related mortality, while Metabolic Associated Fatty Liver Disease (MAFLD) is going to become its most common cause in the next decade. Understanding the complex underlying pathophysiology of MAFLD-related HCC can provide opportunities for successful targeted therapies. Of particular interest in this sequela of hepatopathology is cellular senescence, a complex process characterised by cellular cycle arrest initiated by a variety of endogenous and exogenous cell stressors. A key biological process in establishing and maintaining senescence is oxidative stress, which is present in multiple cellular compartments of steatotic hepatocytes. Oxidative stress-induced cellular senescence can change hepatocyte function and metabolism, and alter, in a paracrine manner, the hepatic microenvironment, enabling disease progression from simple steatosis to inflammation and fibrosis, as well as HCC. The duration of senescence and the cell types it affects can tilt the scale from a tumour-protective self-restricting phenotype to the creator of an oncogenic hepatic milieu. A deeper understanding of the mechanism of the disease can guide the selection of the most appropriate senotherapeutic agent, as well as the optimal timing and cell type targeting for effectively combating HCC.

## 1. Introduction

According to the latest GLOBOCAN report, primary liver malignancies comprised 4.7% of cases of cancer in 2020, but they were also the fourth-leading cause of cancer-related deaths (responsible for 8.3% of total cancer deaths). This suggests that primary liver malignancies are “silent” killers [1]. It is well-established that hepatocellular carcinoma (HCC) is the most frequent primary hepatic carcinoma, representing up to 80% of hepatic malignancies [2]. In comparison to other types of cancer that show slowly declining rates, HCC is steadily on the rise for multiple reasons. While sufficient effort has been put towards establishing effective measures to tackle the rising incidence of HCC, such as rigorous Hepatitis B Virus (HBV) vaccination programmes and wide adoption of Direct-Antiviral Agents (DAA) for Hepatitis C Virus (HCV), much can still be achieved in the primary disease prevention section [2]. 

As evident, the rising numbers in HCC can be partly attributed to an increase in fatty liver disease linked with Metabolic Syndrome (MS) [2]. The gradual adoption of a sedentary lifestyle with a high-fat, high-sugar, low-fibre diet in the last few decades has led to a marked increase in Metabolic Syndrome (MS), an umbrella diagnosis indicative of metabolic dysregulation and insulin resistance, linked with Cardiovascular Disease (CVD), Diabetes Mellitus (DM), and carcinogenesis [3]. 

Fatty infiltration and fibrosis of the liver were observed for the first time by Ludwig et al. in a cohort of patients with a high prevalence of obesity, DM, and normal alcohol consumption, leading to the creation of the term Non-Alcoholic Steatohepatitis (NASH) [4]. This histopathological phenotype evolved into a more easily recognisable clinicopathological entity, Non-Alcoholic Fatty Liver Disease (NAFLD), which was defined by clinical exclusion criteria and solid pathological evidence [5], linked to MS and, subsequently, the sequence of liver steatosis–fibrosis–hepatocarcinogenesis [6]. A recent expert consensus has come to emphasise the pathophysiological importance of metabolic dysregulation and highlight the interaction between MS and hepatic steatogenesis, the initial event in a sequela leading to HCC [7]. In addition to that, the new term, Metabolic-Associated Fatty Liver Disease (MAFLD), offers a definition of the disease that takes into account new diagnostic modalities and facilitates research as well as patient identification [7,8]. The increasing burden of chronic liver disease and HCC attributable to NAFLD/MAFLD is evident in multiple cohorts, especially after the introduction of DAA [9,10,11].

The pathophysiology of hepatocarcinogenesis in the context of MAFLD is not yet fully understood and remains a field of intense research with significant progress over the last few years. Understanding the clinical characteristics of these patients, as well as the pathological features of these tumours, potentially holds the key to deepening our understanding of the intricate mechanisms of disease. A recent meta-analysis showed that when compared to other aetiology HCC, MAFLD–HCC patients are significantly older, have higher Body Mass Index, are more likely to have cardiovascular comorbidities, and are less likely to be cirrhotic [6,12]. When examining tumour characteristics, MALFD patients had higher chances of presenting with uninodular lesions and larger tumour diameter [6,12].

In some high-incidence countries, rigorous surveillance programmes have been introduced and have been proven successful in detecting early disease, amenable to curative modalities of treatment, leading to a notable reduction in disease burden and increased survival rates for patients [2]. However, this is not the case for MAFLD-related HCC, where lower rates of surveillance were reported in comparison to other causes of HCC [12]; nevertheless, that did not result in any significant differences in allocation to curative modalities of treatment versus palliative care [12]. Interestingly, MAFLD-derived HCC patients were less likely to receive a liver transplant because of their multiple medical comorbidities and better-preserved liver function, compared to their hepatitis counterparts [12]. All the above-mentioned facts draw a picture of the natural history of the disease and generate questions about the mechanisms implicated in disease progression. 

There is a rapidly growing body of literature regarding the factors that trigger and the mechanisms involved in MAFLD to HCC progression. Genetic [13,14] and epigenetic factors initiate a constellation of inter- and intracellular mechanisms, amongst which studies have identified autophagy [15,16], ferroptosis [17], gut microbiota [18], lipotoxicity [19], oxidative stress (OS) [20], and cellular senescence (CS) [21]. The last two biological processes, as well as their interplay, seem to hold a key role in the initiation and propagation of fatty liver disease and its sequelae, by harbouring a hostile carcinogenic hepatic microenvironment mediated by different types of CS in the non-linear MAFLD–MASH–HCC sequence of events. The aim of this review is to investigate in depth the role of oxidative stress and cellular senescence, as well as their interaction, in the broad spectrum of MAFLD and its related hepatocarcinogenesis.

## 2. Oxidative Stress-Induced Cellular Senescence in the MAFLD Spectrum: Connected?

### 2.1. Oxidative Stress and Liver Metabolic Disease

#### 2.1.1. The Oxygen Paradox

The appearance of molecular oxygen (O_2_) in the earth’s atmosphere approximately 2.1–2.4 billion years ago led to a change in biodiversity and evolutionary adaptations in living organisms’ metabolism, switching from anaerobic to aerobic [22]. For all aerobic organisms, O_2_ is a key molecule in oxidative energy production, acting as the terminal acceptor of electrons in the final step of the mitochondrial electron transport chain [23]. However, its chemical properties allow for the generation of intermediate by-products of O_2_ metabolism with oxidizing capacity themselves, which can negatively affect cells and, by extension, organisms [23]. Thus, an inconvenient paradox is revealed; O_2_ is indispensable for the survival and growth of aerobic organisms while, in parallel, it has deleterious effects on them [24]. 

#### 2.1.2. ROS in Various Biological Roles

The constellation of molecules that are produced via the incomplete reduction of O_2_ are collectively known as reactive oxygen species (ROS). ROS is a collective term that includes free radicals (species that contain at least one unpaired valency electron), but also some non-radical derivatives of O_2_. “Reactive“ is a relative term, as these molecules display varying oxidative capacity [25]. On one side of the spectrum, molecules such as the superoxide anion (O_2_^•−^) and hydrogen peroxide (H_2_O_2_) are relatively unreactive. On the other side of the spectrum, species such as the hydroxyl radical (•OH), which is generated via the non-enzymatic oxidation of H_2_O_2_, are extremely reactive and can oxidise in a non-discrete manner any biological macromolecule in its close vicinity [23,25]. 

Mitochondria are a major source of intracellular ROS. Within mitochondria, O_2_^•−^ which is produced via the one-electron reduction of O_2,_ is usually the first ROS that is formed. Even though not a strong oxidant, O_2_^•−^ can subsequently produce other downstream ROS. Important sources of endogenous ROS generation are the transmembrane NADPH oxidases. This family of NADPH oxidases includes multiple transmembrane proteins that transport electrons across membranes, using NADPH to allow for the reduction of oxygen to O_2_^•−^. The best-described family member is NADPH oxidase 2 (Nox2), abundant in neutrophils and macrophages, yielding high amounts of ROS that serve for chemotaxis of other phagocytes and dramatic increases in local oxygen consumption, known as the “respiratory” burst [23,26]. The rest Nox enzymes produce less significant amounts of ROS that serve for signalling purposes. Other sites that contribute significantly to the cellular ROS pool are the peroxisomes and the endoplasmic reticulum [23,27]. 

Relative unreactive ROS, with H_2_O_2_ being a prominent example, has been shown to play key roles, directly or indirectly, in redox sensing (a specific molecule sensing the reductive-oxidative state within a cellular component) or redox signalling [25]. A key property of H_2_O_2_ that renders it a suitable molecule for redox signalling is its selective increase of reactivity when encountered with cysteine residues in specific proteins. This means that it can diffuse within a certain distance in a cell without reacting with its surrounding molecules, and then when encountered with these specific cysteine residues, it leads to structural changes of intermediate molecules and activation of signalling transduction pathways [25].

A characteristic example of cysteine-based structural changes linked to redox sensing is the Nrf2/KEAP1 system. Nrf2 (Nuclear factor E2 related factor 2) is a transcription factor that enables the expression of multiple antioxidant enzymes. Keap 1 (Kelch-like ECH-associated protein 1) mediated ubiquitination of Nrf2 under unstressed conditions controls the expression of Nrf2 target genes. However, OS-mediated cysteine residue modifications in Keap1 render it unable to mediate Nrf2 ubiquitination, leading to antioxidant enzymes gene expression [25]. 

#### 2.1.3. ROS Regulation and Removal—Oxidative Eustress and Distress

During their evolutionary course, eukaryotic cells have developed fine-tuned defence mechanisms that can rapidly eliminate the continuously generated O_2_-derived species [28]. A complex and heterogenous assortment of antioxidant enzymes, synthesized by all known aerobic organisms, provide a first line of oxidative defence. As such, superoxide dismutase (SOD) catalyses the dismutation of O_2_^•−^ into O_2_ and H_2_O_2_, while the latter can be safely reduced to H_2_O by catalases (Cat), glutathione peroxidases (Gpx), and peroxiredoxins (Prx) [25,29].

Thus, for normal cellular function to be preserved, a fine equilibrium between the production and elimination of oxidative species has to be maintained. In modern literature, a shift is observed from the classical term “oxidative stress” to two newly described, dynamically evolving states, oxidative “eustress” and “distress” [30]. While oxidative stress reflects an excess of ROS and its consequent macromolecular damage, it overlooks that low steady-state ROS levels regulate several normal physiological functions. The introduction of the terms eustress and distress serves this exact purpose; to clarify that ROS may have both beneficial and harmful effects. Thus, at low physiological levels, ROS plays various physiological roles, and this state is defined as oxidative eustress. Yet, an abundance of ROS is linked with macromolecular damage and cellular dysfunction, and this state is defined as oxidative distress [25,30]. 

The ability of certain ROS to alter macromolecular structures within a cell or cellular compartment, causing structural damage and subsequent dysfunction, was described by Harman in 1956 [31]. In 1972 he expanded his theory: free radicals that are mainly generated in mitochondria as by-products of aerobic metabolism cause oxidative damage. The accumulated oxidative damage to essential macromolecules contributes to aging; the mitochondrial free radical theory of ageing was later renamed oxidative stress theory (OST) [30,32]. The long-term accumulation of faulty macromolecules that cells cannot dispose of leads to disruptions in signalling pathways, as well as a self-perpetuating cycle of metabolic dysregulation and OS, resulting inadvertently in cellular ageing [33,34].

#### 2.1.4. The Role of Iron in OS

An element that is often implicated in redox reactions and oxidative stress regulation is iron. In the human organism, ferrous (Fe^2+^) or ferric (Fe^3+^) iron, either by participating in macromolecular configurations or freely available in small quantities, facilitates a diverse set of cellular functions such as oxygen transport in haemoglobin and myoglobin, cellular respiration, enzymatic reactions, and nucleotide metabolism. The involvement of iron in oxidative stress is classically illustrated in the Fenton reaction, where labile iron, which is normally freely available in the cell in minuscule amounts, catalyses the conversion of H_2_O_2_, a relatively weak ROS, to the •OH. As previously mentioned, the •OH is extremely reactive and can oxidise any macromolecule in its vicinity, leading to temporary—dependent on the available repair mechanisms—or permanent structural and functional damage [22,35].

Furthermore, iron is indirectly implicated in H_2_O_2-_mediated signalling. This link is particularly demonstrated when iron chelation or depletion impacts adhesion molecule expression or transcriptional pathways modulation of lipopolysaccharide exposure response, linking iron with OS and inflammatory response [22]. Labile iron modulation, either by iron-chelating drugs or by diet-derived iron-chelating compounds, was shown to play a key role in preventing H_2_O_2-_mediated apoptosis, and OS and iron metabolism are interconnected and tightly regulated [36,37]. In MAFLD, the dysmetabolic iron overload syndrome, a deranged iron metabolism phenotype, mostly evidenced as hepatocellular or reticuloendothelial iron overload, creates one of the prerequisites for redox imbalance and OS in the context of the disease [6].

#### 2.1.5. OS in Liver Metabolic Disease—The Concept of Lipotoxicity

For a deeper understanding of the role of OS in MS, one must consider the fact that some types of non-adipose cells, such as hepatocytes, β-cells, myocytes, and podocytes, display a certain capacity of storing lipids in their cytoplasm. Oversaturation of these cell types with lipids and their derivatives leads to a generalised metabolic dysfunction with multiple sequelae, termed lipotoxicity [19]. In the case of MAFLD, the hepatocyte is the main site of lipotoxicity, and while the lipotoxic effects of fatty acids are mediated by multiple biological processes, of particular interest is OS. 

The connection between OS and lipotoxicity can be traced to lipid peroxidation. Under OS conditions, polyunsaturated fatty acids can undergo peroxidation. The enzymatic pathway of lipid peroxidation is mediated by different enzymes, amongst which prominent is the role of iron-containing lipoxygenases (LOX), which produce lipid peroxides (LOOH) [38]. Glutathione peroxidase 4 (Gxp4) is the key antioxidant enzyme in neutralising these lipid peroxides [39]. 

However, polyunsaturated fatty acids can also undergo non-enzymatic iron-mediated oxidation, the highly reactive end-products of which are lipid alkoxy free radicals (LO^•^) [38,39]. Their oxidative potential is such that they can create a local self-sustaining milieu of lipid peroxidation, which in its turn, when interacting with other molecules, can lead to neo-epitopes, known as oxidation-specific epitopes, that activate the innate immune system, propagating OS and inflammation and perpetuating chronic degenerative diseases, such as atheromatosis [40]. 

An interesting biological process that could be involved in MAFLD pathogenesis is ferroptosis. Ferroptosis, a recently recognised type of regulated cell death, is elicited by the iron-dependent uncontrolled lipid peroxidation of polyunsaturated fatty acids to lethal levels when Gxp4 is disabled secondary to glutathione depletion. As MAFLD is characterised by antioxidant depletion, iron metabolism dysregulation, and a rich free fatty acid cellular environment, it comes as no surprise that ferroptosis has been shown to contribute to the progression of simple steatosis to steatohepatitis both in vitro and in vivo [41,42,43]. 

When looking outside the hepatocyte, another aspect of MAFLD that implicates OS in its pathogenesis and disease progression is the altered gut microbiota. The changes in gut microbiota in MAFLD patients are attributed to the Western-type diet and insulin resistance. The resultant qualitative and quantitative changes in gut flora create an environment of dysbiosis that leads to an increased baseline inflammatory response of the resident immune cells, such as the hepatic Kupffer cells, which in turn show phenotypes of increased ROS release [44].

### 2.2. Cellular Senescence

#### 2.2.1. Overview of CS

CS, an irreversible state during which cells lose their ability to proliferate, is triggered by several endogenous and exogenous stimuli, such as aging, ROS, oncogene expression, and radiation [45]. CS is a true example of the principle of antagonistic pleiotropy; when in younger individuals and precancerous or early-stage cancer, it serves as a tumour suppressive mechanism, whereas in older individuals or established cancer, its unique properties favour cancer cell proliferation. 

There are two main mechanisms of CS, the replicative CS, which is induced normally under aging conditions, where shortening of telomeres leads inevitably to cell cycle arrest, and the stress-induced premature CS, where external or internal factors cause DNA damage, thereby inducing CS pathways independently of telomere length [46]. Both mechanisms activate the same pathway, known as DNA damage response (DDR), which recruits Ataxia Telangiectasia Mutated (ATM) protein kinase to activate its target protein Checkpoint Kinase 2 (CHK2) [47,48]. Subsequently, ATM and CHK2 stabilise p53 resulting in the upregulation of its target protein p21 [48,49]. Both p53 and p21 inhibit the phosphorylation of the Retinoblastoma 1 factor (RB1), which can now bind to the E2F transcription factor, subsequently preventing cell cycle progression [48]. 

Another well-described molecular pathway leading to CS is the one mediated by p16, encoded by the CKDN2A locus. p16 serves as another proliferation inhibitor, which interferes with the action of Cyclin-dependent kinase (CDK)-4/6 on the Rb proteins [45]. 

These two major CS pathways can be simultaneously activated depending on the cause of DNA damage and the cell type. The belated activation of p16 after the p53/p21 pathway may signify the transition from an early, potentially reversible cell cycle arrest status to an irreversible one [50]. Despite the above two being the best-described mechanisms of CS, the list of molecular mechanisms that participate in the initiation and propagation of CS is still expanding. 

Apart from activating p53 and p16 to promote cell cycle arrest, DDR is also a well-known activator of senescence-associated secretory phenotype (SASP), thus senescent cells, despite entering a cell proliferation arrest, remain metabolically active and are able to communicate with their microenvironment [48]. SASP is composed of several cytokines secreted from senescent cells, such as interleukins (IL-1b, IL-6, IL-8, ROS, growth factors, such as hepatocyte growth factor (HGF), and proteases, such as Matrix Metalloproteinases (MMPs) [51,52]. SASP serves as a regulator of the cellular microenvironment, with autocrine and paracrine actions, by promoting immunological removal of senescent cells, reinforcing senescence in neighbouring cells, and paradoxically acquiring pro-tumorigenic properties [53] depending on the stimulus that initially triggered CS [54]. 

#### 2.2.2. CS and Molecular Players

Although the detection and study of senescent cells are based on several morphological and biochemical features, none of them is completely indicative of CS; thus, a combination of characteristics is typically used to identify senescent cells [55].

Despite the fact that several phenotypical changes have been observed in senescent cells, such as an enlarged and flattened shape, reorganisation of chromatin, and changes in the nuclear membrane [56], these morphological features are not sufficient to detect CS. To that end, numerous studies have underlined specific biomarkers for the identification of senescent cells. 

Proteins playing a crucial role in the induction of cell cycle arrest, such as p53/p21 and p16, are common senescence biomarkers [55]. Furthermore, CS is often characterised by increased lysosomal activity, which can be detected by specific enzymes, such as senescence-associated β-galactosidase (SA-β-Gal), which constitutes a widely known senescence biomarker [57]. Moreover, another established CS biomarker is lipofuscin, a yellowish-brown residue known as “age pigment”, consisting of incompletely degraded oxidized molecules due to increased lysosomal content [58]. Interestingly, it has been demonstrated that SA-β-Gal co-localizes with lipofuscin; thus, these two molecules can be used alternatively to detect CS [59]. An additional CS biomarker is the senescence-associated heterochromatin foci (SAHF), a group of heterochromatin and proteins that suppress the expression of proliferation genes [60]. Some studies have also investigated the detection of SASP compounds, mainly associated with pro-inflammatory features of senescent cells; however, confirming CS based on these factors is still a matter of debate [55].

### 2.3. MAFLD Specific Considerations

#### 2.3.1. CS in MAFLD

There is substantial evidence indicating that CS, especially of the hepatocytes, may foster fat accumulation and inflammation in different stages of MAFLD [58]. In this context, numerous studies have indulged in the role of CS in MAFLD progression, with major CS phenotypical characteristics, such as shorter telomeres and elevated CS biomarkers, such as p53 and SAHF, being observed in patients with MAFLD [61,62].

An increased prevalence of MAFLD in older patients has set the context for further investigation of the role of CS in liver fat accumulation and propagation of the disease [63]. However, as mentioned above, induction of CS is not only mediated by aging and telomere shortening, but also under stressful stimuli provoking DNA damage. 

Preclinical studies in animal models have pointed out a strong correlation between CS and mitochondrial dysfunction, a key parameter of metabolic dysregulation at the early stages of liver steatosis [64]. Moreover, mitochondrial dysfunction results in excessive production of ROS, thereby suggesting a relation between oxidative stress and induction of CS [65]. In animal models of diet-induced MAFLD, propagation of the disease has been shown to progress slower in p53-deficient mice, thus pointing out that the induction of CS favours MAFLD development [60,66]. Intriguingly, elevated mRNA levels of p21 and p16, as well as decreased levels of p53, have been observed in animal models of MAFLD [60]. Furthermore, one of the key characteristics of the transition from early to advanced stages of MAFLD is the inflammation mediated by the recruitment of macrophages in the liver [66]. In this context, CS has been proposed as a crucial regulator through the secretion of chemokines and cytokines of the SASP secretome, such as IL-6, transforming growth factor-beta (TGF-β), which have been found elevated in mice models [67].

Results from human clinical studies further confirm the hypothesis that CS is strongly associated with MAFLD. The length of telomeres has been found to be shorter in patients with MAFLD compared to healthy ones [68]. Furthermore, MAFLD hepatocytes have been found to express high levels of major CS biomarkers, such as p21, and present senescent-associated morphological features, such as larger nuclei. Interestingly, the increased expression of p21 in hepatocytes has been with the degree of fibrosis, irrespective of telomere length, suggesting a premature rather than replicative type of CS [68]. In addition, mutations in the genes coding for the p21 protein have been shown to affect the initial stages of steatosis, but they did not seem to alter the progression of the disease after the establishment of fibrosis, suggesting potentially different pathophysiological mechanisms underlying each stage [69].

Given the complexity of hepatic architecture and function, as well as the broad phenotypic spectrum of MAFLD, one should consider how CS of other cell types helps establish MAFLD. The main regulator of CS in the hepatic microenvironment is hepatocyte-derived SASP, which depending on the duration of the pathological stimulus, can display a vast array of paracrine functions, activating, recruiting, or rendering senescent, adjacent, and distant cells. 

The first cellular type to look into are hepatic stellate cells (HSCs), composite myofibroblast precursors, which during normal liver function, remain in a quiescent state. When a liver injury of any aetiology, including hepatocyte lipotoxicity, is sustained, HSCs enter an activated state, causing liver fibrosis [70]. A recent “proof of concept” study by Wijayasiri et al. showed that hepatocyte CS in MAFLD, evidenced by p16 expression in human MAFLD biopsies with various degrees of fibrosis, is correlated with HSC activation, shown with α-Smooth Muscle Actin (α-SMA), in a positive manner [71]. Furthermore, HSC cultivated in media where senescent HepG2 cells had previously developed showed markers of activation. Interestingly, the only SASP component identified to play a role in HSC activation was the Platelet-Derived Growth Factor (PDGF), both in cultures and cirrhotic patient serum samples [71].

When entering a senescent state, HSCs cease their fibrotic activity and generate a SASP characterised by matrix metalloproteinases, leading to fibrosis restriction and resolution [70,72]. Such an example is stellate cell CS mediated by Insulin Growth Factor–I (IGF–I). Reversal of insulin resistance is linked with higher IGF–I, which induces activated HSC senescence using the p53 pathway both in vivo and in vitro [73,74]. In p53 knockout mice, activated HSC cannot enter CS, with a continuous liver fibrotic phenotype. Interestingly, apart from attenuating fibrosis, another crucial element of CS was observed in this study, immunosurveillance, as senescent HSCs were cleared by NK cells [75]. Last, IL-22, via STAT3, was shown to activate p53/p21 CS in HSC, leading to fibrosis attenuation [76].

Another significant player in this biological process is hepatic macrophages, consisting of two broad categories: Kupffer cells, which are tissue-resident macrophages, and monocyte-derived macrophages [70]. As shown by Irvine et al., OS-induced hepatocyte CS triggers the generation of a pro-inflammatory SASP, including amongst many IL-6 and IL-8, and when macrophages were cultured in a senescent HepG2 medium, inflammatory-type macrophages showed migration activity [77]. Activated macrophages create the inflammatory milieu of NASH. One of the inflammatory cytokines secreted by these activated macrophages, IL-17, has been shown to activate HSCs in a TGF-β1 dependent manner, promoting hepatic fibrosis [78].

#### 2.3.2. CS and OS in MAFLD

The intertwined roles of OS and CS in extrahepatic MAFLD pathogenesis could not be better demonstrated than in the murine model of Sriram et al. Visceral adipose tissue-derived stem cells display high OS characteristics, with intense Nox activity linked to early CS phenotype, attenuated by ascorbic acid treatment, a potent antioxidant [79]. Knowing the well-established connection between visceral adipose tissue and fatty liver disease, the above study shows the importance of extrahepatic CS in MAFLD [80].

In another murine mode developed by Keshavjee et al., reduced protein intake in pregnant mice led to higher amounts of visceral adiposity, MAFLD, and insulin resistance in adult male mice offspring [81]. This MS phenotype was only observed in male individuals and was linked with a variety of premature senescence markers, such as p21 and p16, in the context of a high OS environment (high superoxide anion levels, DNA damages, decreased Cu/Zn SOD, increased catalase protein expression, increased nfr2 and decreased keap1 mRNA expression) [81]. Furthermore, Kondo et al. developed another mouse model that shows SMP-30/SOD1 double knockout individuals developed OS-related MAFLD, possibly secondary to OS-impaired VLDL (very low-density lipoprotein) transport [82]. Ogrodnik and colleagues [83] showed that hepatocyte CS induction is a key event in initiating fat accumulation and correlated it with mitochondrial-derived OS, demonstrating a known observation of Passos et al. [84] in fibroblasts, to be confirmed in hepatocytes. Their key difference is that while Passos showed a p21-dependent CS pathway activation linked to OS, Ogrodnik’s team deployed senolytics to demonstrate a p16 related CS pathway.

Lohr and colleagues also used a C57BL/6J mouse model to study the effects of a high-fat diet and age on MAFLD development. They analysed hepatic mitochondria and concluded that susceptibility to diet-induced obesity and fatty liver increased with age. This also correlated with an age-related reduction in mitochondrial mass and was aggravated by impaired fatty acid oxidation in high-fat diet mice [85].

Kondo et al. examined Senescence Marker Protein–30 (SMP-30)—an antioxidant and anti-apoptotic protein that declines with increasing age—and observed that when comparing standard fat diet-fed mice with SMP-30 knockout to normal counterparts, the former developed fatty liver with inflammatory cells infiltrates and increased oxidative stress linked with impaired fatty acid oxidation and lipoprotein uptake [86]. However, human hepatic biopsy studies of SMP-30 failed to draw conclusive results on the exact role of this ageing-related protein in MAFLD, despite the clear correlation between its expression and disease occurrence [87].

#### 2.3.3. Mitochondrial Dysfunction as a Link between OS and CS in MAFLD

Given the intense oxidative activity that normally takes place within the mitochondria, when the hepatocyte is oversaturated with fatty acids, as in the case of MAFLD, initially, β-oxidation is accelerated [88,89]. When β-oxidation is intensified, mitochondrial ROS production increases dramatically. Fatty acids in the inner mitochondrial membrane are rapidly turned into lipid peroxides, intensifying the OS milieu and leading to macromolecular impairment (mitochondrial DNA, lipids, and proteins) [90]. This functional impairment, in turn, deteriorates the already oversaturated β-oxidation system, leading to further OS deterioration, oxidative phosphorylation deficiency, and mitochondrial dysfunction-driven CS [83]. A key player in mitochondrial dysfunction appears to be acyl-CoA:lysocardiolipin acyltransferase 1 (ALCAT1), an enzyme responsible for cardiolipin—a key structural mitochondrial membrane phospholipid–remodelling, activated under conditions of lipotoxicity and OS, whose detrimental impacts to the mitochondrial structure are mediated by mitofusin 2 (MFN2) downregulation [90,91].

As the disease progresses from simple steatosis to NASH, the repetition of this vicious lipotoxic circle leads to structural mitochondrial defects, with mitochondrial oedema and loss of the typical inner membrane morphology [92]. NASH deterioration is directly linked to impaired mitophagy, the process of removal of defective mitochondria [93,94]. An important link has been observed between impaired mitophagy and CS, as p53 blocks Parkin from translocating defective mitochondria, resulting in their accumulation and intensifying OS [95]. A shared mechanism potentially linking mitochondrial dysfunction and CS is that of the cyclic GMP-AMP synthase and stimulator of interferon genes (cGAS-STING) pathway, as it serves as a SASP activator in CS [96], and can be activated by free mitochondrial DNA leading to an inflammatory phenotype [93].

Furthermore, mitochondrial damage and increased cell-free mitochondrial DNA have been shown to act as damage associate molecular patterns, activating several inflammatory pathways, amongst which is the NLRP3 inflammasome, leading to IL-1β, a critical SASP component [93]. On the other hand, Wiley et al. showed a distinct mitochondrial dysfunction associated with CS phenotype that is activated through a NAD–AMPK–p53 pathway and results in SASP production without IL-1, implicating mitochondrial sirtuins as culprits in this event sequelae [97].

In total, all the above show a clear connection between OS-induced CS and the initiation and progression of MAFLD to NASH and fibrosis. High-fat hepatocyte concentrations, both dietary and from de novo lipogenesis—propagated by insulin resistance—impair hepatic lipid metabolism and generate high amounts of cytoplasmic and mitochondrial ROS, which in turn cause nuclear and mitochondrial DNA damage, triggering CS and installing an elaborate SASP. This SASP displays both autocrine functions, condemning the cell in a vicious circle of metabolic dysregulation secondary to deteriorating OS, and paracrine functions, remodelling the hepatic microenvironment, giving the microscopic characteristics of the MAFLD spectrum.

### 2.4. Does CS Protect from or Induce Hepatocarcinogenesis in the Context of MAFLD?

The role of CS in the progression of hepatocarcinogenesis is considered pivotal, as it has been shown to have both protective and tumorigenic properties.

CS is widely considered a potent tumour suppressive mechanism since the induction of senescence in premalignant hepatocytes has been shown to limit cancer development. One of the main features of senescent cells that allow for the early restriction of hepatic tumorigenesis is their capacity to control their surroundings via SASP secretion. Premalignant or malignant hepatocytes entering the CS programme secrete SASP factors that activate innate immunity and recruit local and distant macrophages to mediate their own clearance, thus halting the progression to an organized HCC tumour. This mechanism of immune-mediated clearance of senescent cells is widely known as immunosurveillance and is of paramount importance as a barrier to hepatocarcinogenesis [98,99,100]. Several SASP components have been studied, considering their suppressive role in HCC development. More specifically, TGF-β1 has been demonstrated to act in an autocrine manner by inducing ROS generation and perpetuating senescent phenotype in HCC mice cells, thereby leading to a restriction in tumour growth by 75% [101,102].

Despite its repeatedly proven cancer-protective properties, when the hepatic micro-environment is changed, and tumour conditions are developed, CS can, under specific circumstances, usher HCC development. This has been explicitly shown in a series of experiments by Eggert et al., who demonstrated that hepatocyte senescence in tumour-free mice led to the recruitment of immune cells, a process mediated by SASP components, thus resulting in clearance of senescent hepatocytes, a well-known tumour repressing effect [103]. However, when HCC cells were infused in mice with senescent hepatocytes, tumour growth was observed, and further analysis showed that the SASP-mediated immune cells inhibited NK and CD8+ T cells from clearing the cancerous hepatocytes [103]. Another clear example of how the shifts in immunosurveillance can herald hepato-carcinogenesis was shown in the experimental and clinical work of Huang et al. Hepato-cellular CS induced a SASP characterized by IL-6 and IL-8 molecules, with macrophage activation. These macrophages displayed M2 polarization, a characteristic of advanced hepatocarcinogenesis, with pro-inflammatory and fibrotic properties, whereas they did not demonstrate any phagocytotic activity, as M1 macrophages show in early HCC, where they clear away premalignant senescent hepatocytes [104].

A progressive shift to a quantitative increase in hepatocyte senescent phenotype driven by telomeres was observed by Donati et al. [105] when comparing cohorts of MAFLD cirrhotic to HCC patients. They showed a higher prevalence of germline mutations of the telomerase reverse transcriptase (TERT), associated with reduced telomere length in HCC patients versus their cirrhotic counterparts [105]. The role of telomere attrition secondary to TERT dysfunction in early MAFLD versus other aetiologies of chronic liver disease has also been highlighted in other studies [62]. Despite these two studies showing a linear relationship between replicative senescence and the natural history of MAFLD, spontaneous somatic mutations of any of the telomerase components due to oxidative stress can trigger a CS-phenotype and act as a possible mechanism of MAFLD-associated hepatocarcinogenesis. Last, TERT promoter somatic mutations along with 8p loss were shown to be characteristic genomic changes of HCC secondary to MAFLD, frequently accompanied by TP53 as well as SWI/SNF complex constituents (ARID1A and ARID2) mutations, clarifying the landscape of genomic instability in these patients [106]. Thus, it is evident that hepatocellular senescence, when maintained in an advanced HCC milieu, can harbour hepatocarcinogenesis by altering SASP and immune cell signalling.

In addition to the role of senescent hepatocytes in HCC development, it is also worth considering the impact of CS in other cellular populations and its effects on tumour biology. Intriguingly, it is well established that the development of HCC secondary to MAFLD often occurs in the absence of liver cirrhosis or in livers with a low degree of fibrosis [6]. In this context, studies have shown that MAFLD-associated factors, such as obesity, have been linked with hepatic stellate cells (HSCs) senescence and expression of tumour-promoting SASP [21]. Although senescence of HSCs has been considered as a protective mechanism against fibrosis and subsequently to HCC, this seems to be reversed in the case of steatohepatic HCC, as depletion of senescent HSCs prevented HCC development in obese mice [107].

**Figure 1:**
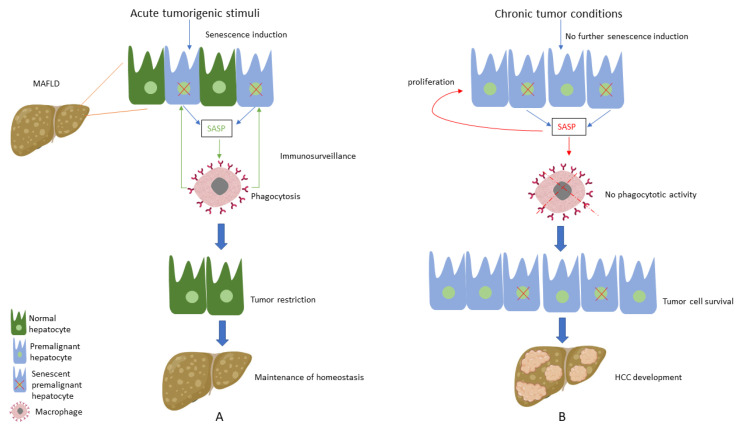
(**A**) Under acute tumorigenic conditions, activation of oncogenes and subsequent DNA damage response results in senescence induction, with senescent hepatocytes secreting multiple types of soluble factors, known as senescence-associated secretory phenotype (SASP). SASP results in the recruitment of immune cells, which eliminate premalignant senescent hepatocytes, a process known as immunosurveillance. Thus, under these conditions, senescence plays a protective role by restricting tumour development and contributing to the maintenance of homeostasis. (**B**) When tumorigenic stimuli become chronic, there is no further senescence induction in hepatocytes. SASP secreted from the existing senescent hepatocytes presents an altered profile by recruiting immune cells, which present no phagocytotic activity, while concomitantly contributing to the proliferation of premalignant cells. Thus, these conditions result in tumour cell survival and the development of hepatocellular carcinoma (HCC).

Considering the above-mentioned pivotal role of CS regarding hepatocarcinogenesis, studies have focused on the factors that trigger this alteration of the CS profile. Interestingly, chronic moderate liver injury, similar to the one MAFLD causes to the liver, led to the development of HCC, while the induction of acute injury in a chronic injury environment activated hepatocellular senescence and subsequently caused HCC reduction [108], showing the detrimental impact of chronic low-intensity stressors in hepatic tumorigenesis. In this context, we suppose that hepatocytes sense initial premalignant stimuli as an acute liver injury during the early stages of HCC development; thus, induction of senescence in premalignant hepatocytes acts as a protective mechanism, leading to the clearance of premalignant cells and tumour restriction [109]. On the other hand, when tumour conditions are established following chronic moderate MAFLD-associated tumorigenic stimuli, senescence is not further activated; thus, premalignant hepatocytes do not enter this cell-cycle arrest process, thereby leading to HCC development and expansion. Furthermore, there is substantial evidence supporting that the existing senescent hepatocytes, instead of promoting immunosurveillance, seem to induce aberrant proliferation of the adjacent hepatocytes via SASP secretion; thus, abnormal proliferation of hepatocytes favours hepatic carcinogenesis [108] (Figure 1).

Collectively, the above demonstrates the importance of cellular senescence in hepatocarcinogenesis. Chronic lipotoxicity and OS induce CS in hepatocytes, triggering SASP secretion, with induction of CS in adjacent cells, such as HSC, halting hepatic fibrosis. Inflammatory phagocytotic cell infiltrates make sure that senescent malignant and premalignant cells are removed. However, continuous chronic exposure to OS lipotoxicity leads to the establishment of a senescent hepatocarcinogenic environment. Local immunomodulatory cells protect malignant hepatocytes, and SASP helps spread CS in neighbouring hepatocytes and HSC. Finally, a chronically ill surrounding hepatic parenchyma helps conserve this carcinogenic niche.

## 3. Future Directions and Conclusions

Overall, there seems to be a connection between oxidative stress and hepatocarcinogenesis. High-fat, high-sugar, and low-fibre diets, in conjunction with a sedentary lifestyle, induce insulin resistance, leading to hepatocyte fatty acid accumulation. This lipid overload, when combined with the iron imbalances observed in MAFLD, creates a lipotoxic OS environment that depletes the antioxidant mechanisms of defence of the cell and propagates macromolecular damage that contributes to a premature CS phenotype. The combination of OS and CS inevitably leads to the progression of plain steatosis to steatohepatitis, and chronicity of these injuries leads to hepatocarcinogenesis, with multiple other mechanisms contributing to that complex pathway.

Given the above-described role of CS in MAFLD-related hepatocarcinogenesis, one could sensibly assume that we could harness the therapeutic potential of CS induction in order to halt the natural history of this spectrum of disease. However, the dual effect of CS on HCC raises several questions on the relevance of senotherapeutics, an umbrella term for all the agents that modify senescent cells in a tissue. First, one needs to consider what intervention would be the most appropriate. The field of senotherapeutics is going through explosive development, and there are several options, from senolytics—agents that selectively facilitate the clearance of senescent cells—to senomorphics—drugs that can inhibit SASP—and senoptotics—that induce apoptosis of CS cells and using cells or extracellular vesicles to induce or reverse a senescent phenotype. The options are endless, and the list is ever-growing [110,111].

However, to yield the desired results, we must clarify the appropriate timing and cell type to target for any of the above categories of senotherapeutics. That is clearly illustrated in the study by Raffaele et al., where the combined use of dasatinib and quercetin, two well-studied senolytics, failed to halt the progression of MAFLD in a high-fat fed and diethylnitrosamine mouse model. Not only that, but the senolytics-treated group of mice displayed a higher ratio of larger HCC foci. The above show that non-targeted senotherapeutic interventions might yield undesired results. Understanding deeply the mechanisms of actions of these agents, as well as the cellular populations we target with them is critical to senotherapeutics success. This could be the impact of senescent macrophages, and impaired immune clearance of cancerous cells [112].

Another question is whether these medications can be used as neoadjuvant therapies before hepatic resection, as bridging agents before liver transplantation, post-resection as adjuvant therapies, or as a treatment for recurrent or advanced disease. As suggested by Giannakoulis et al., senotherapeutics might actually play a key role in tackling sorafenib resistance, and a combination of senescence induction of malignant cells and subsequent clearance with senolytics can be a feasible strategy in treating advanced HCC. However, there is no clinical evidence focusing on MAFLD-related HCC, and most of the current data are drawn from experimental studies [111].

A different pathway for translating our current knowledge of the above-described mechanisms in HCC therapeutics is to tap into iron depletion. HCC patients with low storage of iron showed improved outcomes when treated with sorafenib, while in vitro, the iron-depleting agent deferasirox enhanced the anti-proliferative properties of sorafenib [113,114].

MAFLD is a composite term engulfing pathologic entities ranging from simple steatosis to inflammation and fibrosis that does not follow a linear temporal evolution and can progress or regress, ultimately leading to hepatocarcinogenesis in a substantial number of patients. Interestingly, CS of different resident hepatic cell types as a dynamic cellular process comes as a consequence of chronic exposure to the multiple stressors of metabolic dysregulation, ranging from lipotoxicity to microbial dysbiosis. The alterations that CS causes to the hepatic microenvironment reflect the different pathological stages of MAFLD, as well as the clinical features of these patients.

However, despite the recent emergence of the term MAFLD, giving a clearer and more mechanistic definition of disease, all the above-included studies were designed in the era of NAFLD, carrying its inherent definition limitations. Not only will MAFLD help better identify patients that need therapy or surveillance, but it will help increase sample size in human studies, providing a deeper understanding of the unique role of CS in human HCC. There are still multiple burning questions in the field of metabolic-associated hepatocarcinogenesis. Intensive research in both disease mechanisms and senotherapeutics is still needed in order to draw concrete conclusions as to what the role of CS is during this complex aspect of disease and how it can be harnessed appropriately.

## Data Availability

Not applicable.
